# Comfort and experience with online learning: trends over nine years and associations with knowledge

**DOI:** 10.1186/1472-6920-14-128

**Published:** 2014-07-01

**Authors:** David A Cook, Warren G Thompson

**Affiliations:** 1 Mayo Clinic Online Learning, Mayo Clinic College of Medicine, Rochester, MN, USA; 2 Division of General Internal Medicine, Mayo Clinic College of Medicine, Rochester, MN, USA; 3 Division of Preventive Medicine, Mayo Clinic College of Medicine, Rochester, MN, USA

## Abstract

**Background:**

Some evidence suggests that attitude toward computer-based instruction is an important determinant of success in online learning. We sought to determine how comfort using computers and perceptions of prior online learning experiences have changed over the past decade, and how these associate with learning outcomes.

**Methods:**

Each year from 2003–2011 we conducted a prospective trial of online learning. As part of each year’s study, we asked medicine residents about their comfort using computers and if their previous experiences with online learning were favorable. We assessed knowledge using a multiple-choice test. We used regression to analyze associations and changes over time.

**Results:**

371 internal medicine and family medicine residents participated. Neither comfort with computers nor perceptions of prior online learning experiences showed a significant change across years (p > 0.61), with mean comfort rating 3.96 (maximum 5 = very comfortable) and mean experience rating 4.42 (maximum 6 = strongly agree [favorable]). Comfort showed no significant association with knowledge scores (p = 0.39) but perceptions of prior experiences did, with a 1.56% rise in knowledge score for a 1-point rise in experience score (p = 0.02). Correlations among comfort, perceptions of prior experiences, and number of prior experiences were all small and not statistically significant.

**Conclusions:**

Comfort with computers and perceptions of prior experience with online learning remained stable over nine years. Prior good experiences (but not comfort with computers) demonstrated a modest association with knowledge outcomes, suggesting that prior course satisfaction may influence subsequent learning.

## Background

Given the widespread acceptance and adoption of computer-based instruction over the past decade, it is useful to consider how learners’ attitudes toward this technology have changed over time. Models of technology acceptance suggest that use is predicted by perceived ease of use and perceived usefulness [1,2]. Prior research in health professions education has evaluated attitudes towards computers and computer-based learning primarily among medical students and nurses [3-6] with few studies focused on resident trainees [7]. Moreover, the only study to look at trends over time (among medical students) is now a decade old [8]. It seems reasonable to revisit the question of how comfortable medical trainees are in using computers for learning (i.e., perceived ease of use), how they perceive their online learning experiences (i.e., perceived usefulness), and how these perceptions have changed over time. We might expect that ease of use of online learning would improve over time (as technology improves, learners become more adept, and instructors apply features of effective design), whereas perceived usefulness might remain stable or even decline (as improvements in instructional design are offset by fading infatuation with the new technology and rising expectations).

While computer skills and attitudes are important in their own right, it would also be useful to know how these learner characteristics impact learning in a computer-based course [9]. For example, students who are uncomfortable using computers might find it difficult to learn using computers. By contrast, those with prior experience with online learning might find it easier to learn using computers again. Also, since self-efficacy influences learning [10], those with prior favorable experiences might have greater success in a similar course. Such findings would have important implications for educators regarding how best to support students in an online course: if these student characteristics are associated with learning success, then this would suggest a need for specific support for students with less comfort or experience. Alternatively, if comfort and prior experiences do not systematically influence learning, then it supports the use of online learning across a diverse group of learners. While studies evaluating the association between online learning course ratings and learning outcomes are not uncommon [11,12], studies evaluating the association between pre-course perceptions are fewer and older. One study in health professions education found a modest positive correlation between attitudes toward computer-assisted instruction and course performance [13], while two other studies found essentially no correlation [4,14]. We found no studies examining the relationship between prior experiences with online learning and learning outcomes in a subsequent course. Given the paucity and age of prior research, further investigation seems warranted.

A better understanding of how comfort with computers and perceived usefulness have changed over time would inform arguments favoring or opposed to increased use of computers in education. In addition, knowing the relationship between such ratings and actual learning outcomes would clarify the importance of learner perceptions about their instruction, and inform the need for instructional supports as suggested above. The goals of this study were to determine how postgraduate trainees’ comfort with computers and online learning have changed over the past decade, and how these associate with learning outcomes.

## Methods

Every year from 2003–2011 we conducted a prospective trial of online learning amongst internal medicine and family medicine residents (for example, Cook et al. [15]). Each year residents completed four online learning modules selected from 12 rotating core medicine topics (e.g., diabetes mellitus, hypertension, asthma). The course instructional design varied from year to year, adapting to the research question and incorporating instructional improvement identified in earlier years. Common course elements included text, graphics, links to online journal articles and other resources, and (in nearly all modules) self-assessment questions. Each study was reviewed by the Mayo Institutional Review Board, and all participants provided consent.

### Outcome measures

We asked two questions based loosely on the technology acceptance model’s [2] determinants of use: perceived ease of use and perceived usefulness. Each year we asked, “How comfortable are you in using computers to access the Internet?” (5-point scale: very uncomfortable – very comfortable). From 2004 onward we also asked those who reported previous experience with online learning for agreement with the statement, “My experience with online learning was a good one.” (6-point scale: strongly disagree – strongly agree).

We assessed knowledge using a knowledge test (primarily multiple-choice questions, with a few extended-matching questions) at the end of each online module (11 to 23 questions per module; 53 to 79 questions per year). We also administered this test as a pretest prior to about one-fourth of the modules, sometimes to all participants [15] and sometimes to half the residents [16] depending on the study design. When measured, the pretest-posttest score change ranged 11–22 percentage points (an 11-41% improvement from baseline). In this study, we use only the posttest scores. We followed a rigorous development and pilot testing procedure [15] to ensure that questions reflected the desired objectives and content (content validity evidence). We reused old questions with each three-year cycle, using test item analysis to edit, delete, or replace questions as needed. Reliability of each test was acceptable (Cronbach’s alpha >0.70 in nearly all instances).

### Analysis

We limited data to first-time respondents, to avoid the error of non-independent repeated measures within individuals. We used general linear model regression (SAS 9.3) to evaluate changes over time in average comfort/experience scores. For each study year we calculated the mean knowledge posttest percent correct across all modules, and then used general linear model regression again to evaluate associations between comfort/experience and knowledge posttest scores after adjusting for study year. (Although we compared two or more different instructional formats in most study years, we did not adjust for format differences in the latter analysis because the between-format differences in knowledge scores were small [less than 4%], and because a single unweighted mean score for each individual was deemed satisfactory for the question of interest). We used Spearman’s rho to calculate correlation between ratings.

## Results

**Figure 1:**
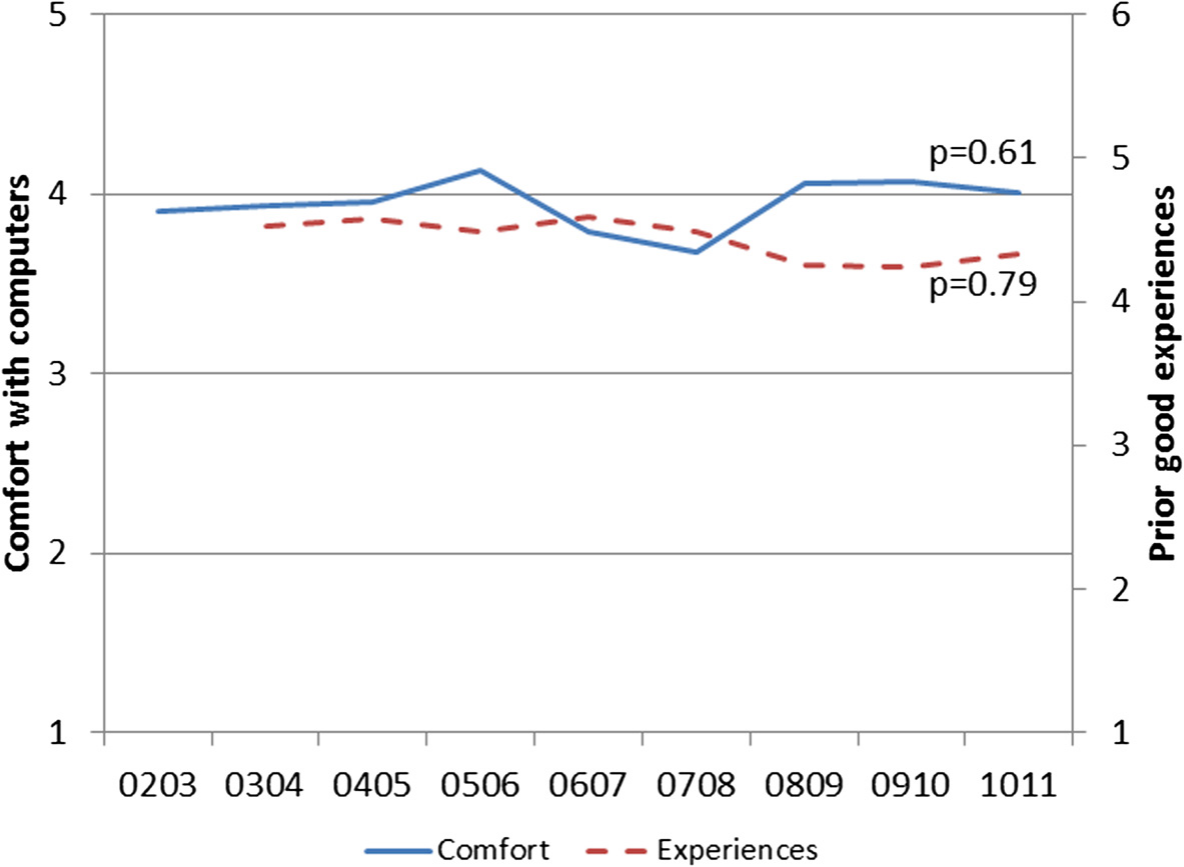
Ratings of comfort and experiences with computers, over time.

**Figure 2:**
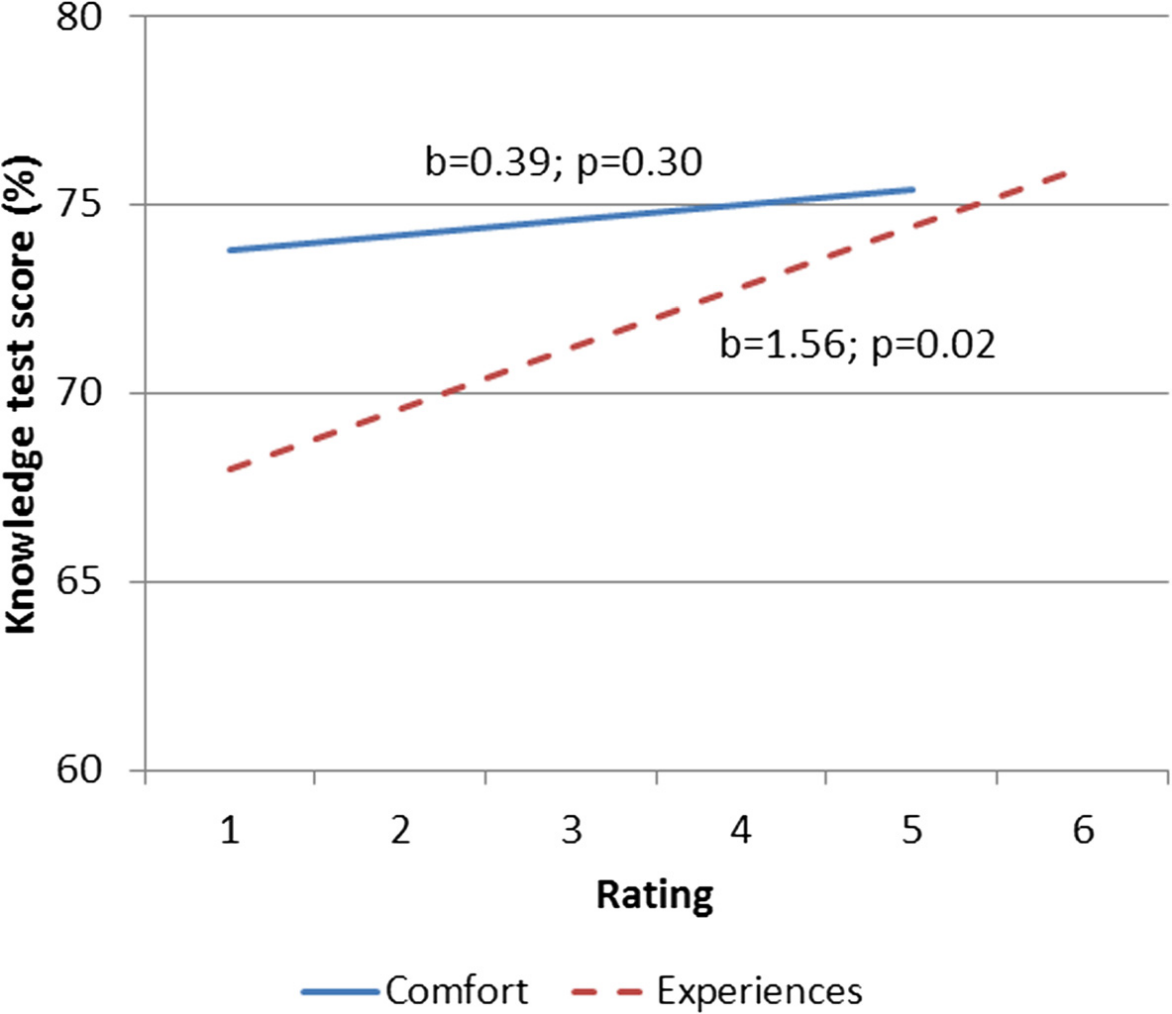
**Associations between comfort and experiences with computers and knowledge posttest scores.** Ratings for comfort and experiences with computers are shown on the x axis; knowledge posttest scores are shown on the y axis.

Over nine years we enrolled 341 unique internal medicine residents and 30 family medicine residents. Of these, 313 (84%) were in their first postgraduate year. Eighty-seven respondents (24%) reported no prior online learning activities; we excluded these from analyses related to prior online experiences.As shown in Figure 1, comfort with computers showed no significant change across years (p = 0.61), with mean across all years 3.96 (maximum 5; range of annual means 3.68-4.13). Likewise, perceptions of prior online learning experiences varied little over time (p = 0.79), with mean 4.42 (maximum 6; range 4.25-4.60). Gender and specialty had no significant influence on either analysis.As shown in Figure 2, comfort with computers showed no significant association with knowledge scores (b = 0.39; p = 0.30). By contrast, perceptions of prior online experiences showed a significant association (b = 1.56; p = 0.02), indicating a 1.56% rise in knowledge score for a 1-point rise in experience rating. Again, gender and specialty had no significant influence on these associations.

We also explored the correlation among the number of prior online learning experiences, the perception of those experiences, and comfort with computers. All of these correlations were small and not statistically significant (p > 0.26), as follows: comfort and perception of prior experience, rho = 0.07; comfort and number of prior experiences, rho = −0.02; rating and number of prior experiences, rho = 0.04.

## Discussion

We found a modest positive association between perceptions of prior online learning experiences and knowledge outcomes measured at the end of an online course. We cannot determine the direction of this relationship (favorable perceptions might enhance self-efficacy and thereby improve performance, or higher-performing students might maintain more favorable perceptions of their education). We also cannot be sure that the relationship is causal, since another influential variable such as ability might influence both outcomes. However, if the effect is causal, it suggests that learners carry forward to new courses their impressions of prior experiences using a given modality. We found no such association for comfort with computers.

Comfort with computers and perceptions of prior online learning experiences remained relatively stable among medicine residents over nine years. We might have expected comfort to rise over time as computers and the Internet became more ubiquitous, but our data suggest that computer literacy may have reached a steady state early in the decade. Viewed through the lens of the technology acceptance model [1,2] we could conclude that learners are rather willing to accept online learning, but not necessarily more willing (or more demanding) than they were at the start of the decade. While some have proposed that those in the rising “Net Generation” have greater skills in and expectations for online instruction, recent evidence suggests that age cohort has less influence than predicted [17,18] and that students might prefer to use computers less than they actually do [19].

We might hope that evolving theory, evidence, and practical knowledge in online learning would lead to evidence-based improvements in course design, and that these would translate to an improved learner experience. However, the stability over time of perceptions of prior online learning experiences suggests either a failure to incorporate such design improvements, or rising student expectations. Limited research does suggest that enhanced course design is associated with improved satisfaction [20], and a worsening of attitudes towards computer-based learning has been observed following exposure to poor course design [3,21]. Our findings suggest the need for improved instructional design in online courses.

Finally, we found no evidence of association between comfort with computers and the number or perception of prior online experiences. One interpretation is that learner perceptions are not systematically influenced for better or worse by comfort or by course participation per se, but rather that individual online courses engender differing perceptions (some more favorable than others). This is consistent with evidence showing substantial variety in both the design [22] and learning outcomes [23] of online courses. Although many studies have shown that satisfaction within a course is associated with learning outcomes, and attitudes toward online learning have occasionally demonstrated associations with course performance [13], we believe our study to be the first to examine the relationship between experiences with online learning and learning outcomes in a subsequent course.

### Limitations and strengths

We asked only two questions in the brief attitude survey, both with limited validity evidence; a more robust development and validation process, or use of an existing scale, would have enhanced the defensibility of these scores. Comfort using computers to access the Internet may be different than comfort for other purposes such as learning. To increase the sample size we used knowledge posttest scores rather than pre-post change. Strengths include the longitudinal design and the use of both attitude and knowledge outcomes. The ratings for comfort or prior experience would not reflect our online course or the specific topics addressed, since data were collected before the first module and we included data only from first-time participants. In fact, since the vast majority of participants were in their first postgraduate year, and most trained at other medical schools, these ratings reflect a broad (albeit not systematic) sampling of institutions.

## Conclusions

Health professions learners appear ready to accept and use online learning. Prior online learning experiences, but not comfort with computers, demonstrates a modest association with knowledge outcomes in online learning. If good experiences indeed beget subsequent learning success, it suggests that even “low level” outcomes like course satisfaction and usability are important to measure and maximize.

## Competing interests

The authors declare that they have no financial or non-financial competing interests.

## Authors’ contributions

DAC and WGT planned the study. DAC analyzed data and drafted manuscript. DAC and WGT jointly revised the manuscript and both approved the final version.

## Pre-publication history

The pre-publication history for this paper can be accessed here:

http://www.biomedcentral.com/1472-6920/14/128/prepub
